# Trained Immunity in Host Defense: Mechanisms, Pathogen Interactions, and Therapeutic Potential

**DOI:** 10.3390/pathogens15080834

**Published:** 2026-08-10

**Authors:** Farha Siddiqui, Anindita Datta, Shivani Choubey, Subhasish Bhadra, Arif Siddiqui, Kalpana Sadawarte

**Affiliations:** 1 Department of Microbiology, People’s College of Medical Sciences & Research Centre, Bhopal 462037, Madhya Pradesh, India; 2 Department of Microbiology, All India Institute of Medical Sciences, Guwahati, Guwahati 781101, Assam, India; 3 Department of Microbiology, LN Medical College and Research Center and JK Hospital, Bhopal 462042, Madhya Pradesh, India; 4 Department of Microbiology, All India Institute of Medical Sciences, Deoghar, Deoghar 814152, Jharkhand, India; 5 Department of Pediatrics, King George’s Medical University, Lucknow 226003, Uttar Pradesh, India

**Keywords:** epigenetic reprogramming, innate immune memory, heterologous immunity

## Abstract

Trained immunity refers to the functional reprogramming of innate immune cells—such as monocytes, macrophages, and natural killer cells—those results in a modulated, and often heightened, response to secondary stimuli. This phenomenon challenges the traditional view that immunological memory is restricted to the adaptive immune system and has emerged as a concept linking host defense, vaccination, and inflammatory disease. This review synthesizes current evidence on the molecular basis of trained immunity, including chromatin remodeling, histone modifications, and metabolic rewiring, and examines how bacteria, viruses, and fungi induce, evade, or exploit these programs. The dual contribution of trained immunity to protective host defense and to immunopathology is discussed, along with its implications for vaccine design—including heterologous protection reported after BCG vaccination—and its potential as a therapeutic target in infectious and inflammatory disease. Key gaps, particularly the duration and reversibility of the trained state, the distinction between trained immunity and related processes such as innate tolerance, and the need for validated biomarkers, are highlighted, together with priorities for future translational research.

## 1. Introduction

Innate immunity has traditionally been observed as a rapid but non-specific first line of defense, in contrast to antigen-specific, memory-forming adaptive responses mediated by T and B cells [1,2]. This view has been revised by the recognition that innate immune cells can undergo functional reprogramming after microbial or sterile activation, enabling a more vigorous response to subsequent, often unrelated, challenges—a process termed “trained immunity” [3]. Unlike adaptive immunity, trained immunity is not antigen-specific; it arises from widespread modification in cellular metabolism and transcriptional potential that broadly increase sensitivity to secondary stimulation [1].

Early experimental models demonstrated that prior exposure to fungal β-glucan or *Candida albicans* increased resistance to reinfection without the need of adaptive lymphocytes [4,5,6]. Subsequent human studies extended this concept, showing that Bacillus Calmette–Guérin (BCG) vaccination can boost cytokine responses to unrelated pathogens, with some studies reporting its persistent heterologous protective effects [7,8]. These findings established trained immunity as a conserved host-defense strategy with relevance extending beyond classical infection biology [7].

The mechanisms underlying trained immunity include pattern-recognition receptor signaling, chromatin remodeling, metabolic rewiring, and reprogramming of hematopoietic stem and progenitor cells (HSPCs) [9,10]. The effects of an initial stimulus can persist for weeks to months, as these changes can also be imprinted at the level of HSPCs, despite the short lifespan of circulating monocytes [11,12]. A synthesis of this evidence is timely, given the rapidly expanding literature connecting trained immunity to host–pathogen interactions, vaccination, and disease process [13,14].

However, existing reviews on trained immunity have largely addressed either its molecular mechanisms or its clinical applications in isolation. The present review aims to integrate the cellular, epigenetic, metabolic, host–pathogen, and translational dimensions of trained immunity within a single framework, with particular emphasis on distinguishing correlative associations from mechanistically or clinically validated findings. In doing so, it seeks to clarify where the evidence base is strong, where it remains preliminary, and where trained immunity intersects most directly with pathogen biology and vaccine design.

## 2. Materials and Methods

This article is a comprehensive review; it is not a systematic review or meta-analysis, and no PRISMA framework was applied. The literature search was conducted in PubMed and Scopus for articles published between January 2012 and June 2025, using combinations of the terms “trained immunity,” “innate immune memory,” “epigenetic reprogramming,” “immunometabolism,” “BCG,” “β-glucan,” and “host–pathogen interactions.” Foundational mechanistic studies published between 2012 and 2020 were included alongside more recent reviews and primary research from 2021 to 2025 to capture both the origin of the concept and its current state. Only English-language, peer-reviewed publications were considered.

Studies were selected for inclusion if they met one or more of the following criteria: they conceptually or experimentally defined trained immunity [1,2,3]; demonstrated innate memory-like responses following microbial or vaccine exposure [4,5,6,9,10]; identified metabolic, epigenetic, or HSPC-level mechanisms [5,6,7,8,11,12,13,14,15,16,17]; linked trained immunity to disease, vaccination, or host–pathogen interactions [1,9,10,11,12,18,19,20,21,22,23]; or provided translational or therapeutic insight [1,22,23,24,25,26,27,28]. Preference was given to studies providing direct mechanistic evidence (e.g., functional or genetic perturbation experiments) and to human data where available; findings drawn solely from cell-culture or animal models are identified as such in the text. Review articles were used primarily to provide context and synthesis rather than as a substitute for primary evidence, and reference lists of key articles were screened for additional relevant citations not captured by the database search. As this is a comprehensive review rather than a systematic review, a formal PRISMA-style screening count was not tracked; based on the search described above, approximately 30 articles were judged directly relevant to the scope of this review and form the primary evidence base cited throughout, supplemented by additional references identified through citation screening.

## 3. Results

### 3.1. Cellular Basis of Trained Immunity

Monocytes and macrophages are the most extensively studied cell types exhibiting trained immunity. Upon re-stimulation following prior exposure to β-glucan, BCG, or other microbial ligands, these cells produce increased amounts of inflammatory mediators such as TNF, IL-6, and IL-1β [3,5,6,7,8,9]. This form of training called mature-cell training is transient, reflecting the short lifespan of circulating monocytes. Tissue-resident macrophages can also undergo local reprogramming after microbial or sterile stimulation, although the resilience and magnitude of this response vary considerably between organs depending on its internal milieu. This more stable, tissue-embedded reprogramming should be distinguished from the transient training of circulating monocytes [17].

Natural killer (NK) cells display several distinct forms of innate memory-like behavior, including enhanced cytokine response or microbial re-stimulation and, in some contexts, adaptive-like memory driven by activating receptors. These processes are mechanistically distinct from monocyte/macrophage training and should not be conflated, although both may be engaged during vaccination or infection [1,2,3].

A third, longer-lived form—central trained immunity—arises from reprogramming of HSPCs in the bone marrow. Experimental studies show that BCG vaccination and β-D-glucan exposure can remodel HSPCs, promote myelopoiesis, and produce myeloid progeny with increased inflammatory and antimicrobial activity [11,12,15]. This reprogramming connects systemic inflammation, infection history, and myeloid lineage bias for a longer duration as it takes place at the progenitor level, and its effects can outlast the turnover of individual mature cells. The key features of trained immunity across these cellular compartments are summarized in Table 1.

### 3.2. Molecular Mechanisms of Trained Immunity

#### 3.2.1. Epigenetic Reprogramming

Epigenetic reprogramming, a defining feature of trained immunity, persists beyond resolution of the initial stimulus. Genome-wide chromatin studies show that trained monocytes acquire activating histone markers—particularly H3K4me3 and H3K27ac—at promoters and enhancers of inflammatory genes, enabling stronger transcriptional responses upon re-challenge [5,6]. This is reinforced by the formation of latent enhancers: regulatory elements that remain in a primed, low-activity chromatin state after the initial signal resolves, allowing more rapid re-activation of inflammatory gene programs upon re-stimulation [13]. These chromatin changes are not stochastic; they are shaped by upstream signaling through pattern-recognition receptors and transcription factors, including NF-κB, AP-1, and C/EBPβ [5,15].

In addition to chromatin marks at NF-κB/AP-1/C/EBPβ target genes, transcriptional regulators with dual, context-dependent roles also support to shaping the trained phenotype. Besides the canonical Smad2/3 pathway, TGF-β1 activates Smad1/5 signaling in primary human macrophages, resulting in a distinct proatherogenic gene program. This illustrates that not all transcriptional inputs downstream of a single cytokine act unidirectionally toward a pro-inflammatory, protective state [29]. TGF-β1-driven Smad1/5 signaling in human macrophages is further amplified under high-glucose circumstances, suggesting that this branch point is itself sensitive to the metabolic environment [30]. There is also evidence that HSPCs can transmit epigenetic marks to their myeloid progeny, potentially extending inflammatory potential beyond the lifespan of mature effector cells; however, the extent, stability, and functional consequences of this transmission in vivo remain incompletely characterized and should be interpreted cautiously [11,15].

#### 3.2.2. Metabolic Reprogramming

Metabolic adaptation is a central component of trained immunity. One of the earliest mechanistic studies showed that trained monocytes shift from oxidative phosphorylation toward aerobic glycolysis via mTOR- and HIF-1α-dependent signaling [7]. This shift has been most clearly demonstrated in monocyte/macrophage models trained with β-glucan or BCG and should not yet be generalized to all trained cell types or stimuli. This metabolic switch facilitates rapid ATP production and biosynthesis and alters the availability of metabolites that regulate chromatin-modifying enzymes. Acetyl-CoA, succinate, and fumarate act as signaling intermediates linking metabolism to inflammatory transcription: succinate stabilizes HIF-1α and supports IL-1β-associated programs, while fumarate accumulation can inhibit histone demethylases and help to maintain activation of histone marks [8,16].

Glucose availability and glycolytic flux also shape innate antiviral responses. Influenza virus infection drives a Warburg-like shift toward glycolysis in immune cells. This glycolytic reprogramming, together with kinases such as PFK1/PFKFB3 and mTOR-dependent signaling, modulates interferon and pro-inflammatory cytokine output during infection, indicating that metabolic reprogramming is pertinent not only to bacterial and fungal training stimuli but also to antiviral innate immunity [31]. Collectively, these findings indicate that immunometabolism is a significant regulator of trained immunity rather than a mere byproduct of cell activation, although whether it should be regarded as the sole organizing principle of the phenomenon remains debated [7,14].

#### 3.2.3. Integration of Metabolism and Epigenetics

**Figure 1:**
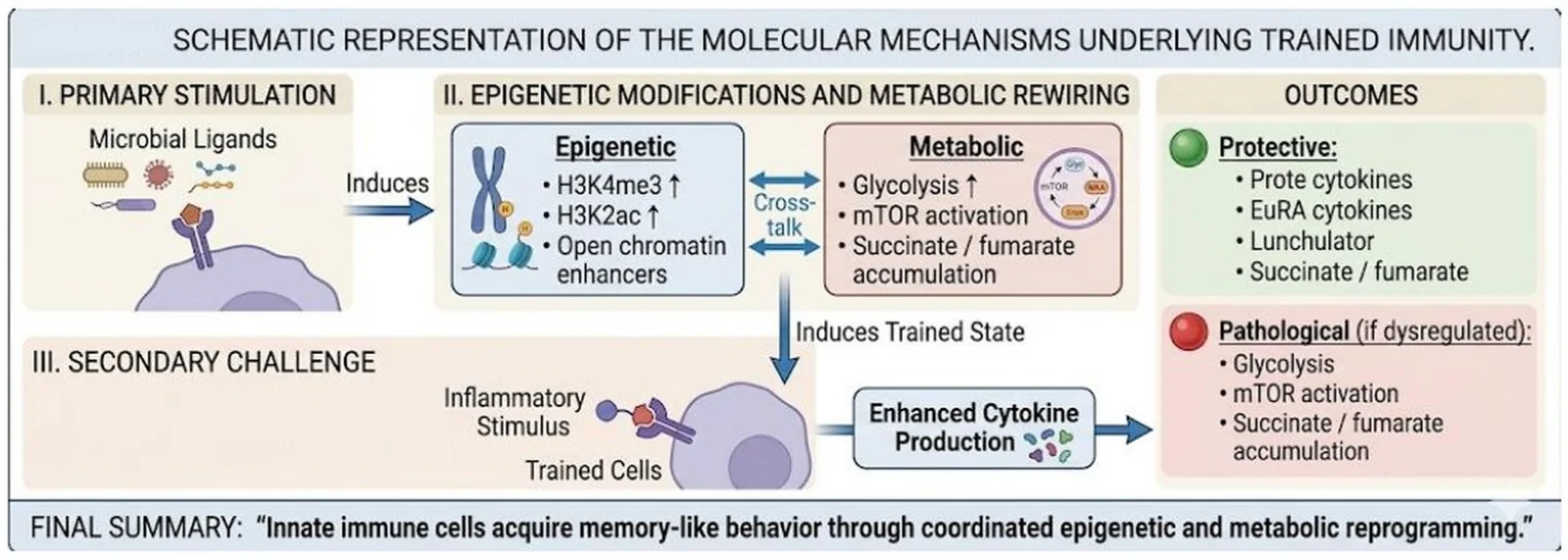
Primary stimulation by microbial ligands induces epigenetic modifications and metabolic rewiring in innate immune cells, resulting in enhanced cytokine production upon secondary challenge.

Available data support a model in which metabolic and epigenetic circuits form a self-reinforcing loop. Epigenetically primed immune genes enhance the potential for inflammatory metabolism, while activation of glycolysis, glutaminolysis, and cholesterol synthesis generates metabolites that directly modify chromatin accessibility [3,18]. This bidirectional relationship has been proposed to explain the persistence and context-dependence of trained immunity. According to recent reviews, the metabolic microenvironment could determine whether training remains tissue-appropriate or becomes maladaptive, and factors like nutrient availability, inflammatory tone, age, and prior infection history may contribute to inter-individual and disease-specific variation [3,17,18]. Much of this integrative model is derived from monocyte/macrophage systems and review-level synthesis rather than from mechanistic studies directly linking specific metabolite–chromatin interactions to functional outcomes across cell types. Original mechanistic studies provide significant insights into these relationships. For example, research has shown that high-glucose culture conditions enhanced macrophage lactate production and phosphofructokinase activity. These metabolic changes specifically stimulate TGF-β1-induced Smad1/5 signaling while having little effect on Smad2/3 signaling. This finding illustrates a concrete mechanism by which increased glycolytic flux can alter transcriptional regulation and shift macrophages toward a more maladaptive, proatherogenic phenotype [30]. The integration of metabolism and epigenetics is depicted in Figure 1.

### 3.3. Protective and Pathological Roles

Trained immunity can potentiate host resistance to infection, complement vaccination, and improve early pathogen containment before adaptive responses are fully mobilized [1,10,13]. However, the outcome of trained immune programming is not simply protective or pathological; it depends on the nature, dose, and timing of the initial stimulus, the tissue and cell type involved, the nature of the secondary challenge, and the underlying condition of the host. Repeated or excessive stimulation does not invariably amplify inflammatory output—in some contexts it can instead induce a state of reduced responsiveness or tolerance, distinct from classical trained immunity, and the factors determining which outcome predominates remain incompletely understood.

**Figure 2:**
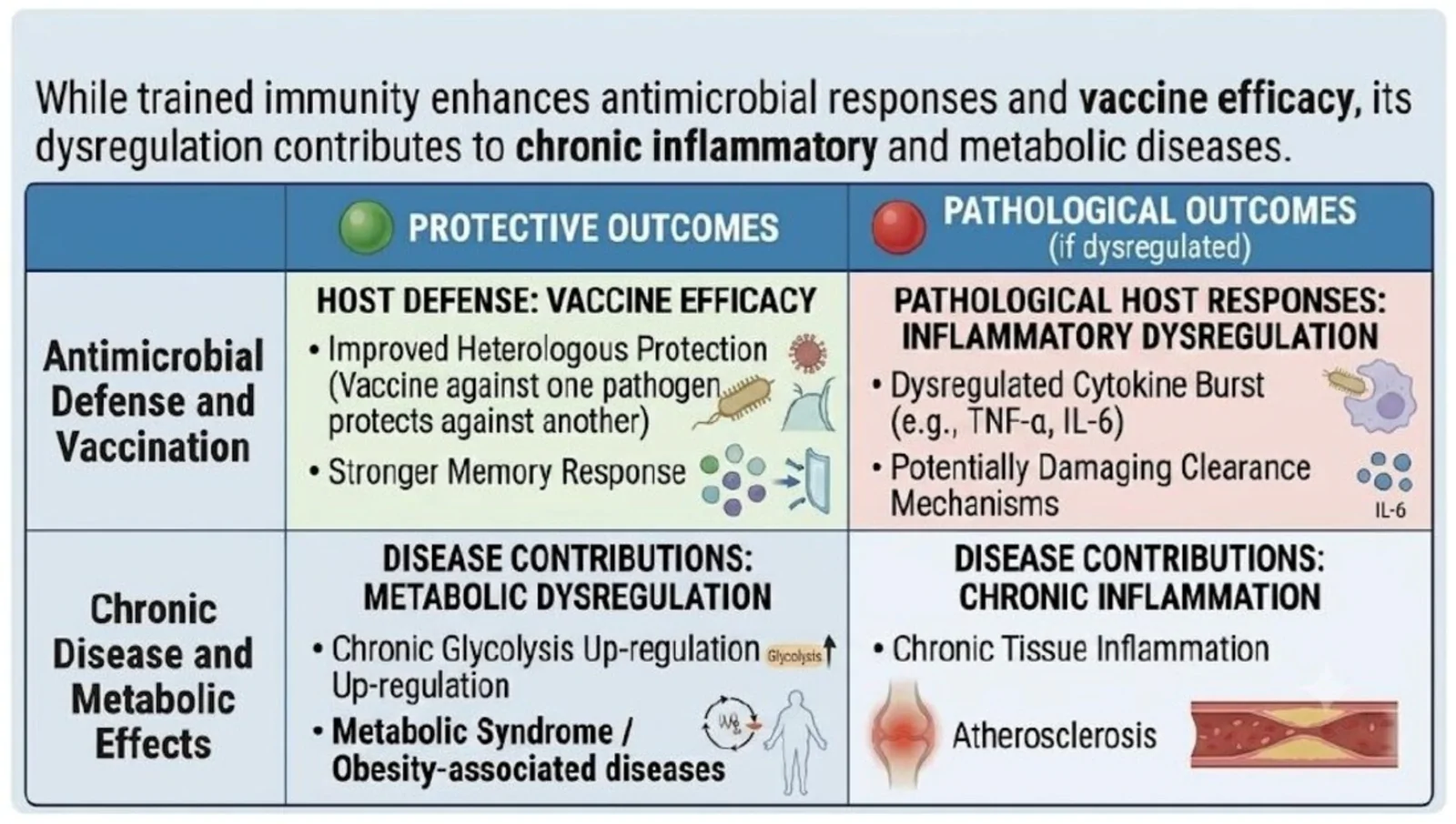
Dual role of trained immunity in host defense and disease. While trained immunity enhances antimicrobial responses and vaccine efficacy, its dysregulation contributes to chronic inflammatory and metabolic disease.

When dysregulated, trained immune pathways have been implicated in metabolic and cardiovascular diseases. Myeloid activation associated with trained immunity can promote plaque formation, endothelial dysfunction, and sterile inflammation [20]. Exposure to a Western diet, has been shown to induce long-lasting innate immune reprogramming, linking nutritional and environmental stimuli to inflammatory memory [21]. This context-dependence is one of the central translational challenges in the field. Therapeutic induction of trained immunity may benefit patients at risk of infection or with impaired vaccine responses, whereas indiscriminate or poorly targeted induction could exacerbate autoimmune, cardiometabolic, or hyperinflammatory diseases [18,20,21,22]. The protective and pathological roles of trained immunity are illustrated in Figure 2.

### 3.4. Trained Immunity in Host–Pathogen Interactions

Trained immunity contributes to resistance against multiple classes of pathogens, but the relationship between microorganisms and trained programs is bidirectional rather than purely host-driven. Fungal β-glucan is the prototypical inducer of antifungal trained immunity, enhancing protection against *Candida albicans* reinfection through monocyte reprogramming [4,6]. Similarly, BCG vaccination induces training that extends beyond antimycobacterial immunity, improving human cytokine responses to unrelated viral infections as per the studies [9]. Conversely, several pathogens appear capable of suppressing, evading, or exploiting trained programs to their own advantage. For instance, by dampening the metabolic or epigenetic changes required for training, or by favoring a tolerant rather than a trained monocyte phenotype following exposure. Although direct mechanistic evidence for pathogen-driven trained immunity in humans remains limited and is largely inferred from in vitro and animal models [19].

Metabolic reprogramming in host cells also differs systematically by pathogen class. In a survey of the literature on carbohydrate metabolism in immune cells across infection types, bacterial and viral infections were most consistently associated with a glycolytic shift in immune cells, whereas fungal and parasitic pathogens more often act by expressing surface effector proteins that modulate host–cell metabolism from outside the cell rather than by triggering the same glycolytic program [32]. This is consistent with the glycolytic and mTOR/HIF-1α-dependent programs engaged by bacterial and fungal ligands such as BCG and β-glucan in monocytes and macrophages [7,9], while viral and parasitic pathogens can additionally alter host glucose and lipid metabolism to favor their own replication or to modulate the antiviral or antiparasitic innate response [31,32]. The extent to which these pathogen-class-specific metabolic effects mechanistically overlap with classical trained-immunity pathways remains an open question warranting dedicated primary research.

**Figure 3:**
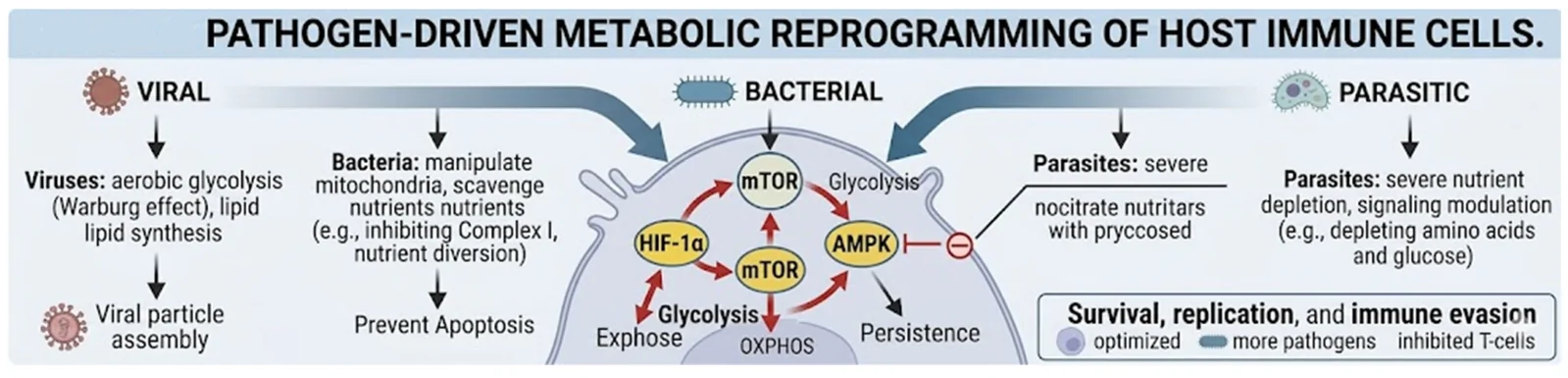
Viruses, bacteria, and parasites manipulate host metabolic pathways to facilitate survival, replication, and immune evasion.

This raises a key pathogen-driven question: does trained immunity consistently benefit the host, or can it, in some contexts, worsen immunopathology or tissue damage? Current evidence suggests a double-edged relationship; short-term reprogramming can accelerate pathogen clearance, whereas prolonged or repeated activation may anchor myeloid cells into a hyperinflammatory state that potentiate disease progression [19]. This balance is particularly relevant in severe infections and chronic inflammatory conditions where microbial exposure and sterile metabolic signals converge [20]. Trained immunity in host–pathogen interactions is depicted in Figure 3.

### 3.5. Trained Immunity and Vaccines

BCG remains the most extensively studied vaccine associated with trained immunity. Human and experimental studies show that BCG can reprogram bone marrow progenitors and enhance ex vivo cytokine responses of innate cells to heterologous stimuli [9,10,11]. It is crucial to distinguish between the well-documented enhanced ex vivo cytokine responses and the clinical protection against heterologous infections that has been demonstrated in some but not all investigations and require to be confirmed in adequately powered clinical trials. Conventional adjuvants may also engage innate signaling pathways, but not all adjuvant effects should be equated with induction of trained immunity in the mechanistic sense described above; this distinction is important when evaluating claims about vaccine platforms.

Beyond conventional vaccines, there is growing interest in vaccines intentionally designed to harness trained immunity. Through the deliberate inclusion of trained-immunity-inducing adjuvants or delivery strategies, especially for immunologically vulnerable populations like neonates and older adults [13,23]. This distinction between incidental trained-immunity effects of existing vaccines and purpose-built trained-immunity-based vaccine platforms is important for pathogen-focused research aiming to broaden protection against emerging infectious threats.

### 3.6. Therapeutic Applications

The therapeutic potential of trained immunity spans three main areas: enhancing host defense, modulating inflammatory disease, and augmenting cancer immunotherapy [1,22,23]. Host-directed strategies that induce trained immunity; using microbial ligands, vaccine-derived signals, or metabolic modulators aim to strengthen innate responses in patients at risk of infection. Conversely, in chronic inflammatory disease, the therapeutic goal is typically the opposite. It inhibits maladaptive training by targeting chromatin-modifying enzymes, cytokine pathways, or metabolic checkpoints [1,20,21,22]. These induction- and inhibition-based strategies rely on distinct mechanistic rationales and should not be conflated.

In oncology, trained immunity inducers may stimulate antitumor responses by reprogramming suppressive myeloid populations, particularly in combination with existing immunotherapy platforms [24]; this remains a largely preclinical area of investigation. Organ-specific differences are also relevant, as the training phenotype in lung, gut, liver, or bone marrow may vary in duration and function [17,25]. Across all of these applications, most evidence remains preclinical or early-clinical stage; translation is further complicated because the same pathways that strengthen antimicrobial defense can also promote collateral inflammation, and patient-specific factors—including age, metabolic status, infection history, and microbiota composition—are likely to influence treatment response [22]. Precision based, stratified approaches will therefore be important for clinical translation.

## 4. Discussion

Trained immunity has reshaped understanding of host defense by demonstrating that innate immune cells and their progenitors can develop long-lasting, memory-like responses after primary activation [1,2,3]. These responses are sustained by coordinated metabolic and epigenetic reprogramming and can stimulate defense against diverse pathogens [5,6,7,8,9,10,11,12]; however, when dysregulated, the same pathways can precipitate chronic inflammation and metabolic diseases [20,21,22].

Several important limitations and uncertainties temper this picture. First, much of the mechanistic evidence for trained immunity derives from in vitro monocyte/macrophage models and short-term murine studies; the extent to which these findings generalize to humans in vivo, over clinically relevant timescales, is less well established. Second, findings from cell culture, animal models, and human clinical studies are not interchangeable: associations between epigenetic or metabolic changes and enhanced cytokine output should not automatically be interpreted as evidence of improved clinical protection, and causal claims require direct functional or interventional evidence rather than correlation alone. Third, trained immunity must be carefully distinguished from related but mechanistically distinct processes, including innate immune tolerance, persistent low-grade activation, and inflammatory memory; conflating these processes risks overstating either the protective or pathological consequences of innate reprogramming. Fourth, the duration and reversibility of the trained state in humans remain unclear, particularly across different tissues and clinical settings, and tissue specificity is likely to be an important but understudied variable, given evidence that training phenotypes differ between organs [17]. Fifth, the field currently lacks validated biomarkers to prospectively identify trained states in patients or to monitor response to therapeutic modulation.

Addressing these gaps will likely require single-cell and multi-omics approaches capable of linking chromatin state, transcriptional output, metabolism, progenitor dynamics, and tissue localization at high resolution [18]. Such integrative analyses could help identify patients most likely to benefit from trained-immunity induction versus those at greater risk of inflammatory harm, supporting more precise, stratified therapeutic strategies. From a pathogen-focused perspective, an important direction for future work is determining how different microorganisms imprint distinct innate programs, how these programs interact with host metabolism, and how they might be safely modulated for prevention and treatment [1,23,27,28]. As a comprehensive review, systematic study selection with quantity and qualitative assessment can have a possibility to get selection bias. Much of the evidence is derived from in vitro and animal studies, limiting direct clinical translation. Furthermore, heterogeneity in experimental models and outcome measures complicates comparisons across studies. The representative studies discussed in this review are summarized in Table 2.

## 5. Conclusions

This review set out to integrate the cellular, molecular, host–pathogen, and translational dimensions of trained immunity while distinguishing well-supported findings from those that remain preliminary. The evidence indicates that innate immune cells and their progenitors can acquire long-lasting, memory-like responses after primary activation, sustained by coordinated metabolic and epigenetic reprogramming, and that these responses can enhance defense against diverse pathogens. At the same time, when dysregulated, the same pathways can contribute to chronic inflammation and metabolic disease. Key uncertainties—including the durability and reversibility of the trained state, its distinction from innate tolerance, tissue-specific variation, and the absence of validated biomarkers—currently limit clinical translation. A balanced perspective is therefore warranted: trained immunity is best understood as a context-dependent host program, with meaningful but still-developing implications for infectious disease biology, vaccine design, and therapeutic development, rather than as a uniformly protective or detrimental process.

## Figures and Tables

**Table 1:** Overview of the biological basis and functional outcome of trained immunity.

Feature	Description	Representative Mediators	Key References
Inducing stimuli	Microbial ligands, live vaccines, or sterile metabolic signals can initiate trained programs	β-glucan, BCG, Western diet-related signals	[4,9,21]
Principal cells	Monocytes, macrophages, NK cells, and HSPCs participate in training, via mechanistically distinct routes	Monocytes/macrophages, NK cells, HSPCs	[1,3,11,12]
Epigenetic basis	Sustained chromatin remodeling increases accessibility of inflammatory genes	H3K4me3, H3K27ac, latent enhancers	[5,6,13,15]
Metabolic basis	Aerobic glycolysis and related pathways support trained responses	mTOR, HIF-1α, fumarate, succinate	[7,8,16]
Functional consequence	Enhanced cytokine output and broad antimicrobial responsiveness on restimulation	TNF, IL-6, IL-1β	[3,5,9]
Long-term maintenance	Bone marrow (HSPC-level) reprogramming sustains central trained immunity	HSPC remodeling, myeloid bias	[11,12,15]
Protective role	May improve resistance to unrelated infections and support vaccine responses	Heterologous effects reported after BCG	[9,10,23]
Pathological role	May promote sterile inflammation and cardiometabolic disease if dysregulated or persistent	Atherosclerosis, diet-induced inflammation	[20,21,22]

**Table 2:** Summary of the representative studies on trained immunity.

S. No	Study	Year	Study Type	Brief Method	Main Finding	Ref.
1	Defining trained immunity and its role in health and disease	2020	Review	Conceptual and mechanistic synthesis	Established the modern framework of trained immunity in health and disease	[1]
2	Trained immunity: a program of innate immune memory in health and disease	2016	Review	Broad synthesis of innate immune memory evidence	Broad synthesis of innate immune memory evidence	[2]
3	Trained immunity: reprogramming innate immunity in health and disease	2021	Review	Mechanistic review of metabolic and epigenetic pathways	Consolidated cellular and molecular basis of trained immunity	[3]
4	*Candida albicans* infection affords protection against reinfection	2012	Experimental	Reinfection model	Demonstrated early evidence for innate memory-like protection	[4]
5	Epigenetic programming of monocyte-to-macrophage differentiation and trained innate immunity	2014	Experimental	Genome-wide epigenetic profiling	Defined chromatin remodeling as a core mechanism	[5]
6	β-glucan reverses LPS-induced tolerance	2016	Experimental	β-glucan training after tolerance induction	Showed that trained immunity can counter innate immune tolerance	[6]
7	BCG vaccination protects against experimental viral infection in humans	2018	Clinical/experimental	Human vaccination and viral challenge model	Demonstrated heterologous antiviral effects of BCG	[7]
8	Long-lasting effects of BCG vaccination	2014	Human mechanistic study	Cytokine response analysis after vaccination	Showed durable nonspecific immune enhancement after BCG	[8]
9	mTOR- and HIF-1α-mediated aerobic glycolysis as metabolic basis for trained immunity	2014	Experimental	Metabolic inhibition and transcriptional analysis	Identified glycolysis as essential for training	[9]
10	Glutaminolysis and fumarate accumulation integrate immunometabolic and epigenetic programs	2016	Experimental	Metabolomics and epigenetic integration	Linked fumarate accumulation to sustained trained responses	[10]
11	BCG educates hematopoietic stem cells to generate protective innate immunity against tuberculosis	2018	Experimental	HSPC analysis after BCG exposure	Showed bone marrow-level programming by BCG	[11]
12	Modulation of myelopoiesis progenitors is an integral component of trained immunity	2018	Experimental	Bone marrow progenitor tracing	Demonstrated trained myelopoiesis as a key mechanism	[12]
13	Latent enhancers activated in immune response	2013	Experimental	Chromatin accessibility mapping	Provided enhancer-based explanation for transcriptional memory	[13]
14	Immunometabolism overview	2016	Review	Synthesis of immune-metabolic crosstalk	Framed metabolism as a regulator of immune function	[14]
15	C/EBPβ-dependent epigenetic reprogramming	2020	Experimental	Stem cell and epigenetic analysis	Explained progenitor-level training mechanisms	[15]
16	Succinate is an inflammatory signal that induces IL-1β through HIF-1α	2013	Experimental	Metabolite signaling analyses	Identified succinate as an inflammatory metabolic signal	[16]
17	Immune gene priming	2019	Experimental	Gene-priming and chromatin profiling	Clarified how immune loci remain poised after stimulation	[17]
18	Trained immunity: priming, tolerance	2022	Review	Integrative mechanistic review	Update the in vivo and in vitro experimental models and human subjects in mechanistic update	[18]
19	Trained immunity and pathogen evasion	2021	Review	Host–pathogen interaction synthesis	Highlighted that pathogens may exploit or evade trained programs	[19]
20	Trained innate immunity as a mechanistic link between sepsis and atherosclerosis	2014	Review/translational	Pathology-focused synthesis	Connected trained immunity with vascular inflammation	[20]
21	Western diet triggers trained immunity	2018	Experimental	Diet-induced inflammatory memory model	Linked sterile metabolic signals to maladaptive training	[21]
22	Therapeutic targeting of trained immunity	2019	Review	Translational and therapeutic synthesis	Outlined druggable pathways in trained immunity	[22]
23	Trained immunity: General and emerging concepts	2024	Review	Molecular mechanism of disease and therapeutic overview	Molecular mechanism of trained immunity with cancer and disease development	[23]
24	The causes and consequences of trained immunity in myeloid cells	2024	Review	Mechanistic and disease-focused review	Detailed drivers and consequences in myeloid cells	[24]
25	Trained immunity inducers in cancer immunotherapy	2024	Review	Cancer immunotherapy analysis	Highlighted translational use in tumor settings	[25]
26	Trained immunity across organs	2024	Commentary/Review	Organ-focused synthesis	Described tissue-specific variation in trained responses	[26]
27	Infection-induced trained immunity: a twist in paradigm of innate host defense and generation of immunological memory	2025	Review	Infection-focused synthesis	Emphasized the double-edged effect of infection-driven training	[27]
28	Metabolic regulation of trained immunity in innate cells	2024	Review	Multi-omics and metabolic synthesis	Updated the metabolic–epigenetic framework	[28]
29	TGF-β1, Activates Smad1/5 Pathway in Primary Human Macrophages and Induces Expression of Proatherogenic Genes		experimental	Disease pathogenesis TGF-β1mediated	TGF-β1, Activates Smad1/5 Pathway in human macrophages	[29]

## Data Availability

Figure 1, Figure 2 and Figure 3 and the graphical abstract were prepared by the authors using Gemini 3.1 pro (Google), an AI-assisted image-generation tool, to render the schematic diagrams summarizing concepts and mechanisms described in the text. No copyrighted, previously published figures were reproduced or adapted. The AI tool was used solely to generate the visual layout of figures; the underlying scientific content, structure, and legends were determined and verified by the authors. No AI tool was used to generate the manuscript text.

## Abbreviations

**Abbreviation**	**Full Form**
AP-1	Activator Protein 1
BCG	Bacillus Calmette–Guérin
C/EBPβ	CCAAT/Enhancer-Binding Protein beta
H3K4me3	Histone H3 lysine 4 trimethylation
H3K27ac	Acetylation of lysine 27 on histone H3
HIF-1α	Hypoxia-Inducible Factor 1-alpha
HSPCs	Hematopoietic Stem and Progenitor Cells
IL-1β	Interleukin-1 beta
IL-6	Interleukin-6
mTOR	Mechanistic (or Mammalian) Target of Rapamycin
NF-κB	Nuclear Factor kappa-light-chain-enhancer of activated B cells
NK cells	Natural killer cells
PFK1	Phosphofructokinase-1
PFKFB3	6-Phosphofructo-2-kinase/Fructose-2,6-bisphosphatase
TGF-β1	Transforming Growth Factor beta 1
TNF	Tumor Necrosis Factor
